# Novel Non-Invasive Diagnosis of Bladder Cancer in Urine Based on Multifunctional Nanoparticles

**DOI:** 10.3389/fcell.2021.813420

**Published:** 2022-01-31

**Authors:** Jinshan Xu, Shuxiong Zeng, Jun Li, Li Gao, Wenjun Le, Xin Huang, Guandan Wang, Bingdi Chen, Zhensheng Zhang, Chuanliang Xu

**Affiliations:** ^1^ Department of Urology, Changhai Hospital, Naval Medical University, Shanghai, China; ^2^ Institute for Regenerative Medicine, Shanghai East Hospital, The Institute for Biomedical Engineering & Nano Science, Tongji University School of Medicine, Shanghai, China; ^3^ Department of Pathology, Changhai Hospital, Naval Medical University, Shanghai, China; ^4^ Department of Nutrition, Changhai Hospital, Naval Medical University, Shanghai, China

**Keywords:** bladder cancer, diagnosis, nanoparticles, urine cytology, nano

## Abstract

**Objectives:** Tumor cells were reported to have perpetual negative surface charges due to elevated glycolysis, and multifunctional nanoprobes (Fe_3_O_4_@SiO_2,_ mNPs) could attach onto tumor cells *via* opposite surface charges. We thus evaluated whether mixing mNPs with urine could improve the sensitivity of urine cytology test (UCT).

**Methods:** We developed a novel UCT method by mixing urine with mNPs (Nano-cytology) to harvest more tumor cells during UCT procedures. The same voided urine sample was divided equally for the Nano-cytology and UCT assay, and evaluated by cytopathologists in a blinded way. The accuracy of UCT, Nano-cytology, and the combination of the two approaches (Nano-UCT) for detecting bladder cancer were determined.

**Results:** Urine samples were prospectively collected from 102 bladder cancer patients and 49 non-cancer participants from June 2020 to February 2021 in Changhai Hospital. Overall sensitivity of the Nano-cytology assay was significantly higher than that of the UCT assay (82.4 vs. 59.8%, *p* < .01). Sensitivity for low- and high-grade tumors was 79.1% and 39.5% (*p* < .01) and 84.7% and 74.6% (*p* = .25) for Nano-cytology and UCT, respectively. Specificity of Nano-cytology was slightly lower than that of UCT (89.8% vs. 100%, *p* = .022), which is mainly caused by severe urinary tract infection. In addition, Nano-UCT showed increased sensitivity with 90.2% for overall patients, and 83.7% and 94.9% for low- and high-grade tumor, respectively.

**Conclusion:** The Nano-cytology assay had a significantly improved sensitivity compared with UCT for detecting bladder cancer patients. It represents a promising tool for diagnosis of bladder cancer in clinical practice.

## Introduction

Bladder cancer (BC) is the 10th most commonly diagnosed cancer worldwide (International Agency for Research on Cancer, 2021). Approximately 75% of patients with BC present with non-muscle-invasive bladder cancer (NMIBC) (Compérat et al., 2015). Patients with NMIBC have a significant risk of recurrence and progression after transurethral resection of bladder tumor (TURBT) (Babjuk et al., 2019). As a result, patients with NMIBC need lifelong surveillance after therapy. Cystoscopy and urine cytology are the most important examinations for both diagnosis and surveillance of BC. However, cystoscopy is invasive and costly, and may miss flat lesions and carcinoma *in situ* (CIS) with a false-negative outcome ranging from 10% to 40% (Grossman et al., 2005). Currently, although numerous non-invasive urine biomarkers have been developed for diagnosis of UC, urine cytology test (UCT) is the only recommended liquid biopsy for surveillance of BC in different treatment guidelines for BC (Witjes et al., 2021). LCT (liquid-based cytology test) is commonly used in UCT daily practice with the advantages of being specific and non-invasive, but LCT depends on the experience of cytopathologists and the sensitivity is unsatisfactory, varying from 29% to 84% for different grades of BC, especially lower for low-grade BC (Yafi et al., 2015; Babjuk et al., 2022). Therefore, there is a clinical need to decrease the technique difficulty of LCT and improve its sensitivity.

With the rapid development of nanoscience and nanotechnology, numerous studies have focused on exploring nanomaterials for diagnosis and treatment of a myriad of cancers (Sahoo et al., 2007). Although glutamine, sialic acid, etc. can affect the surface charge of tumor cells, the most important effect is the that of glucose metabolism. Elevated glycolysis in tumor cells usually led to a higher-level secretion of lactate, which caused negative charges on the cell surfaces, while the surfaces of normal cells remain charge-neutral or slightly positive due to normal glycolysis (Chen et al., 2016). Taking advantage of the unique biophysical property of tumor cells, novel multifunctional nanoprobes (Fe_3_O_4_@SiO_2_, mNPs) with positive charges were developed to capture tumor cells specifically without using any specific molecular marker (Chen et al., 2016).

In the present study, we aimed to evaluate whether mixing mNPs with urine (Nano-cytology) could help to identify tumor cells in patients with hematuria, and to compare the diagnostic accuracy of Nano-cytology with traditional UCT in a prospective, blinded, single-center clinical trial.

## Materials and Methods

### Construction of Electrically Charged mNPs

The mNPs are composed of the positively charged mNPs and the negatively charged mNPs. Detailed procedures to construct mNPs were described in a previous study, and all the mNPs used in the present study were synthesized by The Institute for Biomedical Engineering & Nano Science, Tongji University School of Medicine (Chen et al., 2016).

### Cell Culture

T921, 5637, EJ, RT-4, Biu-87, and T-24 cells were grown in RPMI 1640 medium supplemented with 10% (v/v) heat-inactivated fetal bovine serum (FBS) and 1% (v/v) penicillin–streptomycin (PS) at 37°C in a 5% CO_2_ humidified atmosphere. Hela cells were cultured at 37°C in DMEM supplemented with 10% FBS and 1% (v/v) PS in a humidified atmosphere in the presence of 5% CO_2_. Non-cancerous bladder epithelial cells SVHUC were grown in F12K supplemented with the 10% (v/v) heat-inactivated fetal bovine serum (FBS) and 1% (v/v) penicillin–streptomycin (PS) at 37°C in a 5% CO_2_ humidified atmosphere. Typically, cells were passaged by trypsinization and maintained in medium accordingly.

### Preparation of Primary Cells

Primary bladder tumor cells were isolated from rates of xenograft bladder cancer models and bladder cancer patients’ tissue who underwent radical cystectomy in our hospital as previously reported (Xu et al., 2013). A total of six cases of tumor tissue and corresponding normal urothelial tissue (adjacent to cancer) were collected from patients. Primary cells were cultured in 24-well plates with 1 × 10^5^ cells per well. Cells were incubated in RPMI 1640 medium containing 10% FBS, 100 U/ml penicillin, and 100 µg/ml streptomycin. The research was approved and performed under the ethical and legal standards of the Ethics Committee of Shanghai Changhai Hospital (CHEC2019082).

### Fluorescence Microscopy Analysis

Bladder tumor cells and Hela cells were grown at 37°C under normal cell culture conditions to 70%–80% confluency. The culture wells were washed thoroughly with PBS, trypsin, and resuspended in PBS. mNPs were added at the indicated concentrations to the cell suspensions and incubated on ice for 5 min with gentle agitation. After incubation, the NP-bound cells were captured by a permanent magnet placed against the side wall of the tube and free cells were removed by washing three times with PBS. The captured cells were released by removing the magnet and resuspended in PBS. Then, the captured cells were imaged using fluorescence microscopy (DMIL, Leica, Germany) and counted by a hemocytometer.

### Patients and Samples

During June 2020 to February 2021, participants were prospectively recruited from Changhai Hospital. The inclusion criteria for patients with bladder cancer were as follows: patients diagnosed with bladder cancer and underwent TURBT or cystectomy for treatment; male or female ages >18 years; patients without any other tumor history; and patients who signed the informed consent form. The inclusion criteria for participants in the control group were as follows: participants without any tumor disease and willing to provide urine samples, participants who signed the informed consent, and participants with any benign urinary diseases, such as urinary stones, cystitis glandularis, interstitial cystitis, and cystitis. Exclusion criteria included patients unwilling to sign the consent form or unwilling to provide urine sample for analysis; patients who already had an indwelling catheter; and patients with incidental prostate cancer by prostate biopsy, transurethral resection of prostate, or cystectomy. Urine samples of approximately 100 ml were collected from participants at the time of hospital admission. Urine samples were divided equally for UCT and nano-cytology analysis.

### Urine Cytology Test

Approximately 50 ml of urine was used for UCT analysis. We used the BD-TriPath Preparatory (BD-TriPath Imaging, Burlington, NC), a liquid-based sampling technique, for the preparation of urine cytologic samples. Slides were prepared and processed according to the manufacturer’s protocol, and were evaluated by experienced cytopathologists blinded to the patients’ clinical information. The UCT assay was given as negative (normal cells and atypical cells were regarded as negative) and positive (suspicious tumor cells and tumor cells were regarded as positive).

### Nano-Cytology Assay

**FIGURE 1:**
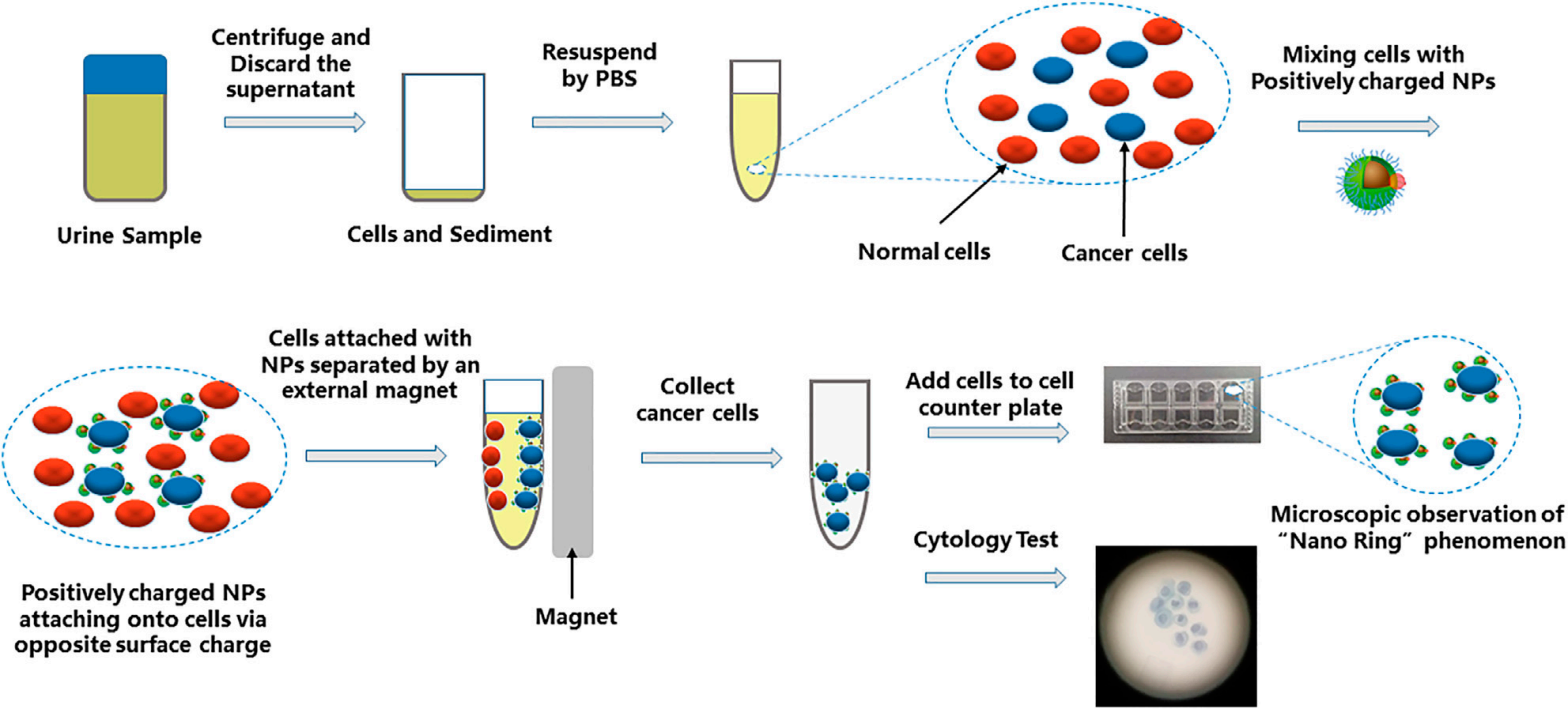
Schematic diagram showing the procedure of Nano-cytology. The cells in suspension are mixed with positively charged NPs. Cell/NP bindings take place due to the opposite charges. The captured cells are magnetically attracted to the side of the tube by a permanent magnet. The “Nano-ring” phenomenon can be observed by a microscope. The collected cells also need a cytology test.

Urine samples (50 ml) were processed for the Nano-cytology assay as soon as they were obtained. Urine was centrifuged at 600 *g* for 5 min. The cell pellet was resuspended in phosphate-buffered saline (×1) and centrifuged at 600 *g* for 5 min. Cells seeded in a 5-ml centrifuge tube were incubated with the positively or negatively charged mNPs at 4°C for 15 min. After incubation, the magnet was used to capture the cells surrounded by mNPs. All cell-mNPs incubations were performed on ice to avoid endocytic events. The captured cells were resuspended in 200 µl of phosphate-buffered saline (×1), and 20 µl of the remixed samples was placed in the cell counting plate and imaged using microscopy (Cellometer Auto 1000, Nexcelom Bioscience, United States) to look for suspicious tumor cells. If suspicious tumor cell was found, the result score value = 1; otherwise, value = 0. The rest of the captured cells were prepared and processed according to the UCT protocol (Figure 1). Cytology was evaluated by experienced cytopathologists blinded to the patients’ clinical information. The results were given as negative (N) or positive (P). Finally, a Nano-cytology modality was developed combining the value and the cytology result. The protocol could be made as follows: positive results include P1, P0, and N1; a negative result is N0.

### Statistical Analysis

Sensitivity, specificity, negative predictive value (NPV), and positive predictive value (PPV) were calculated for Nano-cytology, UCT, and a combination of these two methods (Nano-UCT). Subgroup analyses were performed for different tumor grades and stages. The Fisher’s test (by SPSS Statistics software, v22.0, IBM Institute) was used to determine the statistical difference for categorical variables. A *p*-value <.05 was considered statistically significant.

## Results

### Interaction of the mNPs With Bladder Cancer Cell

**FIGURE 2:**
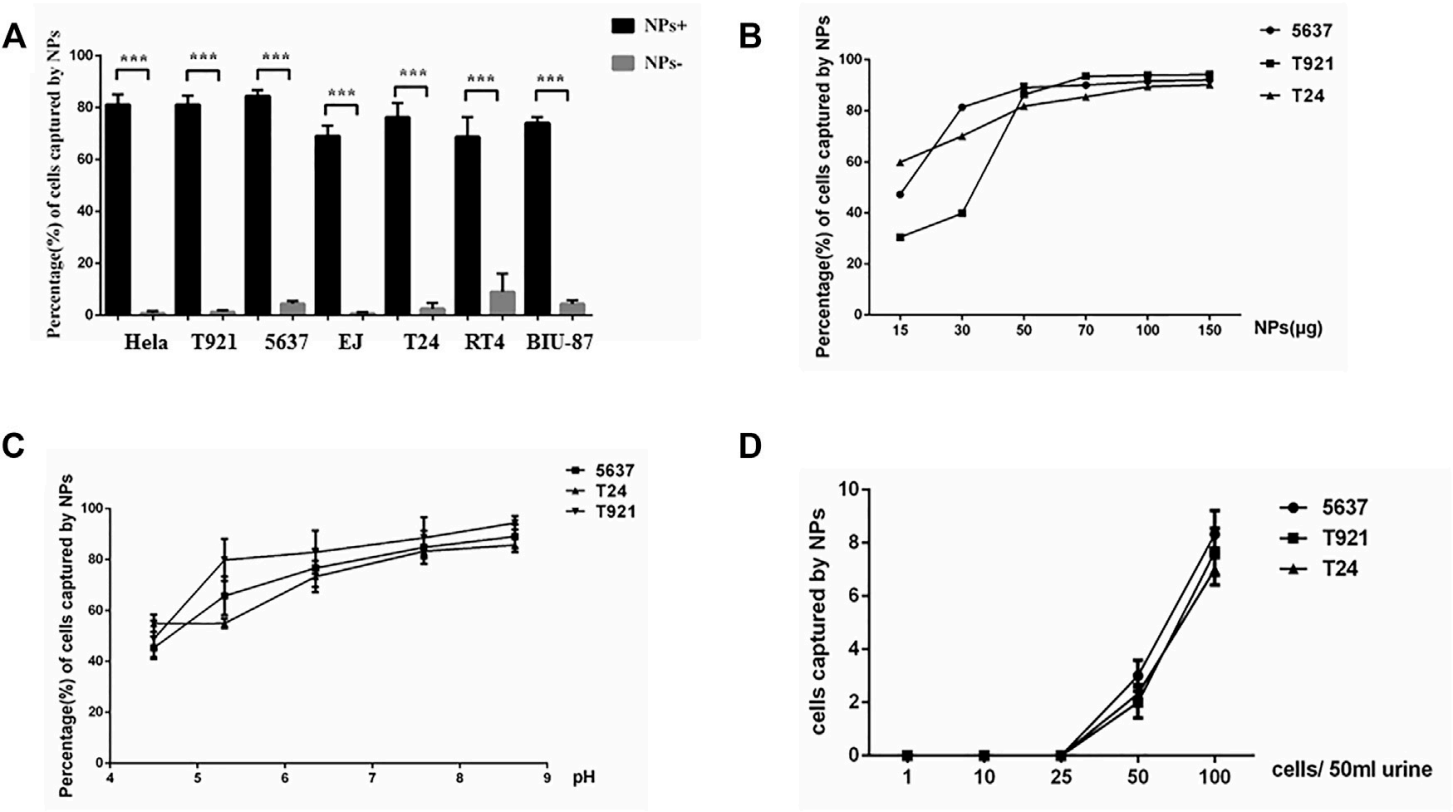
Interaction of the mNPs with bladder tumor cells. **(A)** Positive mNPs and negative mNPs have completely different patterns of interaction with bladder tumor cells. ***p < .001. **(B)** The magnetic capture efficiencies of bladder tumor cells by NPs of positive charges are plotted at various mNP concentrations. **(C)** The magnetic capture efficiencies of bladder tumor cells by mNPs of positive charges are plotted at various pH value. **(D)** Positive mNPs can capture cancer cell if more than 50 cells in 50-ml samples were given.

To determine whether positively charged mNPs could bind bladder tumor cells specifically, six different bladder cancer cell lines and the Hela cell line (positive control) were incubated with positively and negatively charged mNPs, respectively. Upon electrostatic interaction with enough mNPs, the captured cells were “pulled” to the side of the tube by a magnet. Figure 2A shows that positively and negatively charged mNPs had a completely different pattern of interaction with tumor cells, and positively charged mNPs had a significantly higher ability to capture tumor cells, which was in line with our previous study (Chen et al., 2016). Furthermore, we tried to characterize the interaction of the bladder cancer cell and mNPs; a constant number of three different bladder cancer cells were incubated with various concentrations of positively charged mNPs ranging from 5 to 150 µg/ml, and with 50 µg/ml mNPs at different pH. The efficiency of the magnetic capture ability for bladder tumor cells by mNPs is shown in Figures 2B,C; 50 µg/ml positive NPs were enough to capture most of the 5 × 10^5^ bladder cancer cells, and the mNPs showed stable capture efficiency with pH ranging from 6 to 9. Moreover, we demonstrated that 50 µg/ml positively charged mNPs could capture 50 tumor cells per 50 ml of urine (Figure 2D).

### mNPs Formed Nano-Ring Around Bladder Tumor Cells

**FIGURE 3:**
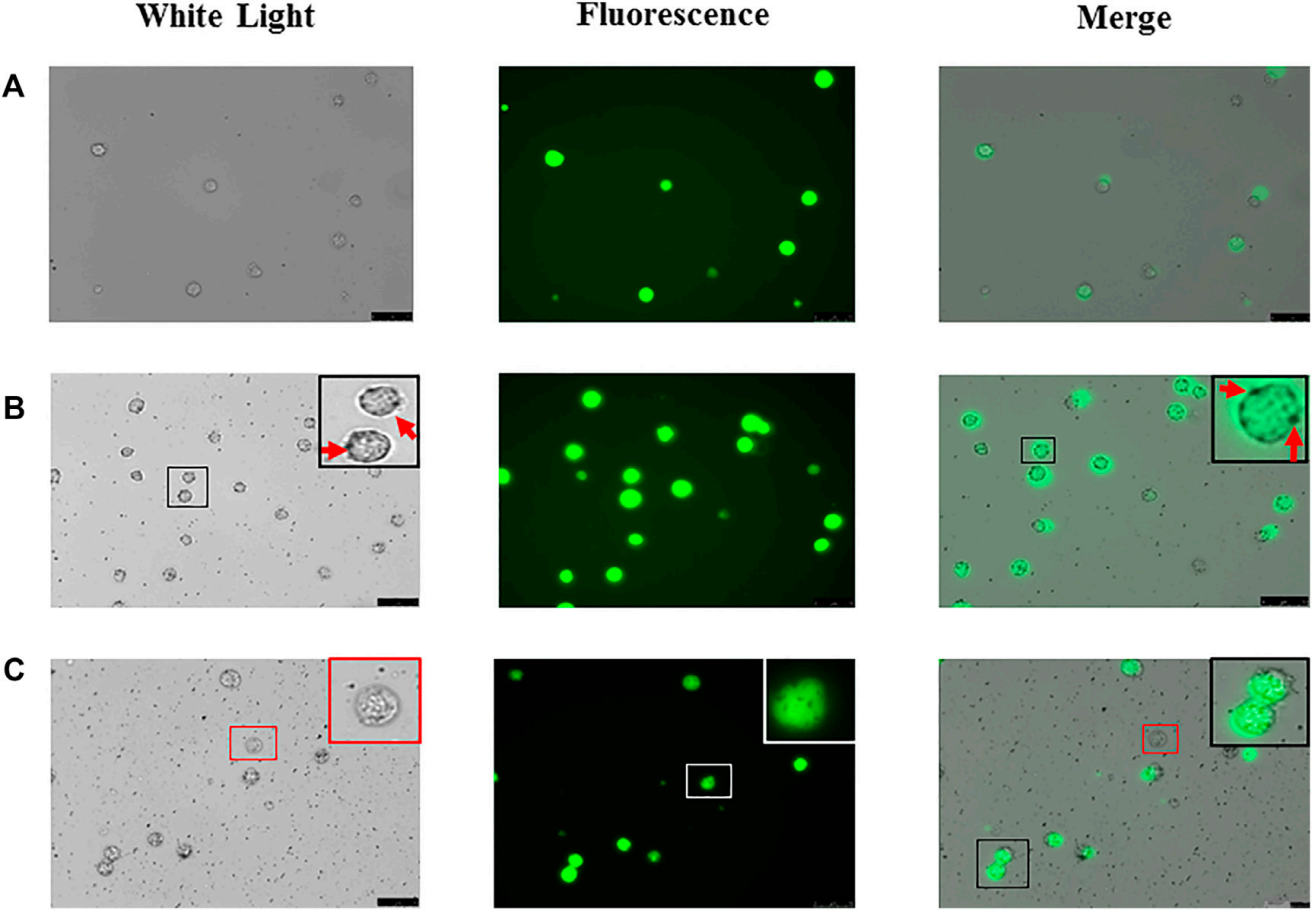
Foundation of “nano ring” in bladder tumor cells. **(A)** Biu-87 cells show green fluorescence. **(B)** Incubate fluorescent Biu-87 cells with positive mNPs, cells were surrounded by the mNPs (red arrows). **(C)** Mix the same number of fluorescent Biu-87 cells, SVHUC cells, and positive mNPs; mNPs only surround green fluorescent Biu-87 cells (black frame), and no NPs surround the only cell without fluorescence (red frame).

We transfected green fluorescent labeled lentivirus into Biu-87 cells (Figure 3A) and incubated with mNPs. As shown in Figure 3B, we found positively charged mNPs binding to and covering the surface of Biu-87 cells. The phenomenon looked like rings (nano-ring) under a microscope (Figure 3A). We further performed an experiment to validate the positively charged mNPs specifically capturing tumor cells. Labeled Biu-87 cells were mixed with the non-cancerous SVHUC cells in a ratio of 1:1 and incubated with positively charged mNPs. As displayed, nano-ring was found around Biu-87 cells, but not detected around SVHUC cells, which showed no fluorescence (Figure 3C).

### The Ability of mNPs to Differentiate Tumor Cells in Primary Cultured Cells

**FIGURE 4:**
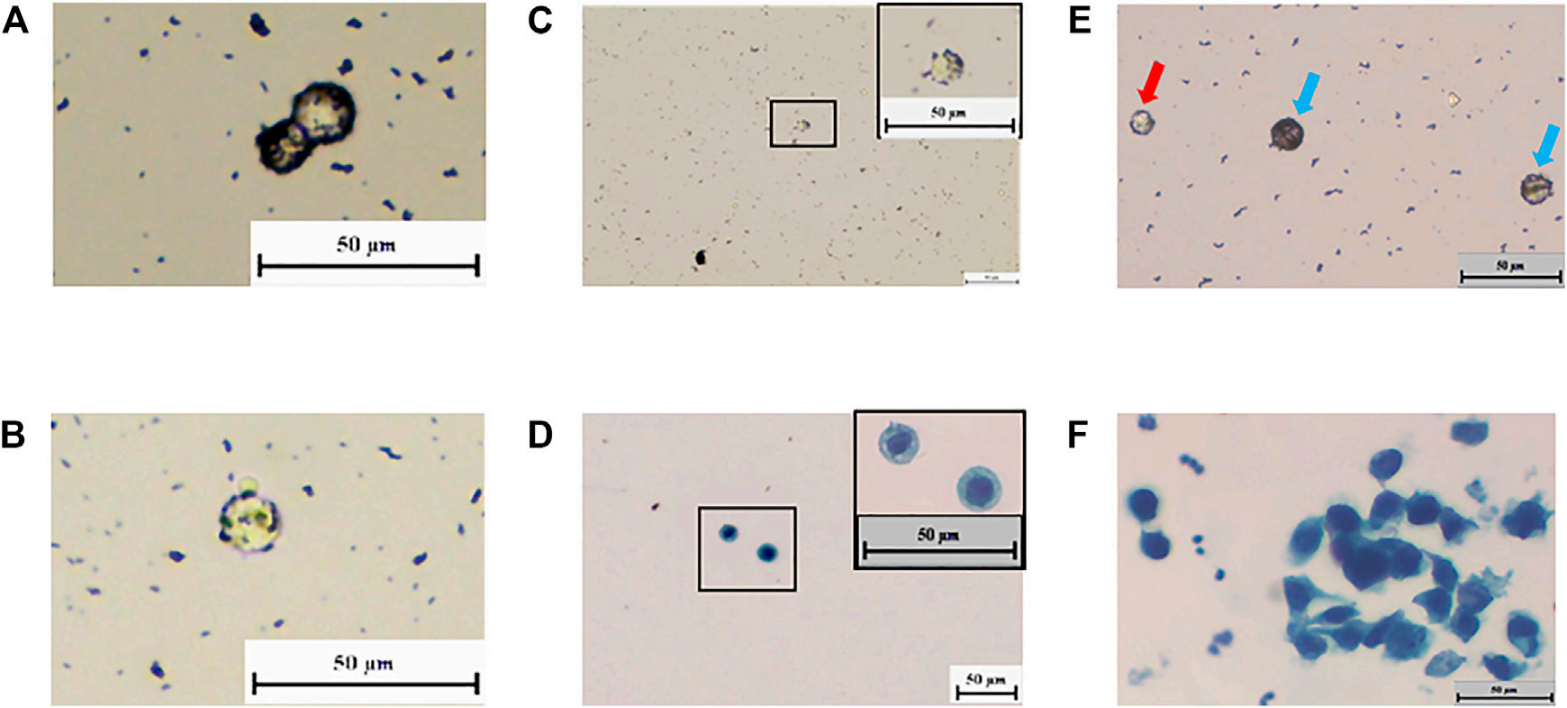
“Nano ring” in living organisms’ bladder tumor cells. **(A–C)** In subcutaneous tumorigenic mice’s cells and patients’ tissue, the “nano ring” phenomenon is obvious. **(D)** Cytology test proves the NP-bound patient’s tissue cells were tumor cells. **(E)** Incubate positive mNPs with Bladder cancer patients’ urine exfoliated cells. “Nano ring” phenomenon is obvious. White blood cell (red arrow) is smaller than the bladder cancer cell (blue arrow). **(F)** Cytology test proves that the NP-bound patients’ exfoliated cells were tumor cells.

To determine whether the nano-ring phenomenon also existed in primary cultured cells, different types of primary cells were harvested from SCID, FVB, C57, nude mice’s bladder, bladder cancer patients’ tissue, and subcutaneous xenograft tumor by T24 cells on nude mice and incubated with positively charged mNPs. No nano-ring phenomenon was found in the normal mice cells. Cells from subcutaneous xenograft tumor and patients’ tumor tissue, the nano-ring phenomena were obvious (Figures 4A–C). The mNPs bond cells were then magnetically captured and separated. Slides contained separated cells that were confirmed to be tumor cells by cytopathologists blinded to the information of cells (Figure 4D).

### The Ability of mNPs to Differentiate Tumor Cells in Urine

We further investigated whether mNPs could identify tumor cells from urine and avoid the influence of red blood cells and white blood cells in urine. Urine exfoliated cells from bladder cancer patients were used to incubate with positively charged mNPs. Many cells showed the nano-ring phenomenon (Figure 4E). In Figure 4E, white blood cells were smaller than bladder cancer cells, and nano-ring was not seen. While red blood cells were found in hematuria cases due to its properties, which adheres to the tube and is hard to wipe off. However, this could be distinguished by the feature of round cake with concave center on both sides. As shown in Figure 4F, tumor cells with nano-ring were detected and confirmed by cytopathologists.

### Nano-Cytology Showed Improved Sensitivity Compared With Urine Cytology Test

One hundred and six patients and 49 controls were enrolled to compare the sensitivity between nano-cytology and traditional UCT assays. Four participants were excluded due to the incidental prostate cancer found after cystectomy. Detailed information of included participants is shown in Table 1. Compared with UCT, nano-cytology identified BC with significantly improved overall sensitivity (82.4% vs. 59.8%, *p* < .01), but slightly lower specificity (89.8% vs. 100%, *p* = .022, Table 2). Nano-cytology resulted in higher sensitivity for identifying low-grade (79.1% vs. 39.5%, *p* < .01) and high-grade BC (84.7% vs. 74.6%, *p* = .25) compared with UCT. Moreover, the advantage of nano-cytology was maintained in the detection of NMIBC (82.4 vs. 56.5%, *p* < .01), but there was no difference between muscle-invasive BC (82.4% vs. 76.5%, *p* = 1). When combining these two methods together, nano-UCT resulted in a sensitivity of 90.2%, a specificity of 89.8%, a PPV of 94.8%, and an NPV of 81.5% (Table 3).

**TABLE 1:** Patient baseline characteristics.

Urine samples	Tumor	Control	*p*-value
Participants included	102	49	
Participants excluded	4		
Gender			.601
Male	87	41	
Female	15	8	
Age			
Mean (SD)	66.51 (12.31)	66.24 (13.55)	.354
Tumor stage			
<pT2	85		
≥pT2	17		
Tumor grade			
Low grade	43		
High grade	59		
Benign urinary diseases in control			
Urinary stones		13	
Benign prostate hyperplasia		21	
Incontinence		4	
Others		11	

**TABLE 2:** Detailed comparison of sensitivity obtained by Nano-cytology and UCT for BCa detection in relation to tumor stages and grades.

Tumors	Nano-cytology		UCT		*p*-value
	*n* Positive/*n* Total	Sensitivity (%)	*n* Positive/*n* Total	Sensitivity (%)	
BC	84/102	82.4	61/102	59.8	.001*
BC by grade					
Low	34/43	79.1	17/43	39.5	.000*
TaLG	27/34	79.4	9/34	26.5	.000*
T1LG	6/7	85.7	6/7	85.7	1
T2–4LG	1/2	50.0	2/2	100.0	.248
High	50/59	84.7	44/59	74.6	.253
TaHG	9/12	75.0	8/12	66.7	.653
T1HG	28/32	87.5	25/32	78.1	.320
T2–4HG	13/15	86.7	11/15	73.3	.361
BC by stage					
<pT2	70/85	82.4	48/85	56.5	.000*
Ta	36/46	78.3	17/46	37.0	.000*
T1	34/39	87.2	31/39	79.5	.362
≥pT2	14/17	82.4	13/17	76.5	1.000
Control	*n* Negative/*n* Total	Specificity (%)	*n* Negative/*n* Total	Specificity (%)	
All	44/49	89.8	49/49	100.0	.022*
Normal	32/32	100.0	32/32	100.0	1
Infection	12/17	70.6	17/17	100.0	.003*
PPV	94.3%		100%		
NPV	71.0%		54.4%		

NPV, negative predictive value; PPV, positive predictive value.

*Significant, *p* < .05.

**TABLE 3:** Detailed comparison of sensitivity obtained by Nano-UCT for BCa detection in relation to tumor stages and grades.

Tumors	Combination of Nano-cytology and UCT	
	*n* Positive/*n* Total	Sensitivity (%)
BC	92/102	90.2
BC by grade		
Low	36/43	83.7
TaLG	27/34	79.4
T1LG	7/7	100.0
T2–4LG	2/2	100.0
High	56/59	94.9
TaHG	12/12	100.0
T1HG	30/32	93.8
T2–4HG	14/15	93.3
BC by stage		
<pT2	76/85	89.4
Ta	39/46	84.8
T1	37/39	94.9
≥pT2	16/17	94.1
Control	*n* Negative/*n* Total	Specificity (%)
All	44/49	89.8
Normal	32/32	100.0
Infection	12/17	70.6
PPV	94.8%	
NPV	81.5%	

NPV, negative predictive value; PPV, positive predictive value.

## Discussion

In the present study, we found that positively charged mNPs could specifically capture bladder tumor cells in urine, and tumor cells that bond with mNPs could be enriched under the influence of a magnet. The patient exfoliated cells that showed the nano-ring phenomenon should be regarded as suspicious tumor cells and tumor cells. The sensitivity of standard UCT for detecting urothelial carcinoma is low, largely due to its inability to process the entire sample, paucicellularity, and the presence of background cells (Miyake et al., 2021). Our novel approach could help to reduce the confounding background cells such as normal urothelial cells, apoptotic cells, and red and white blood cells in urine. Cytopathologists thus could focus on evaluating suspicious cells captured by mNPs. As a result, our study showed that nano-cytology had a significantly higher sensitivity (82.4% vs. 59.8%), especially for low-grade tumor cells (79.1% vs. 39.5%), and comparable specificity compared with traditional UCT. In addition, the mNPs are cost-effective, which can be synthesized at a cost of $100 and test tens of thousands of samples. So there is not much difference between nano-cytology and UCT in terms of cost.

Urine cytology is a commonly used noninvasive approach for detecting tumor cells in the urinary tract with high specificity. Currently, UCT is recommended in the surveillance of high-risk NMIBC in clinical guidelines at certain intervals (Babjuk et al., 2019). However, the sensitivity of UCT varied from 29% to 84% for tumors of different grades and stages (Dimashkieh et al., 2013), and the sensitivity for detecting CIS was 28%–100% (Babjuk et al., 2022). Meanwhile, the accuracy of UCT largely depends on the experience and skill of the technicians and cytopathologists. The Paris System for Reporting Urinary Cytology have been put forward to standardize the reporting system of UCT (Babjuk et al., 2022). Meilleroux et al. (2018) reported that the Paris System helped to characterize the atypical urothelial cells and low-grade urothelial neoplasm. Several novel approaches have been explored to improve the sensitivity of UCT. Birkhahn et al. (2013) invented a novel portable microfiltration device for the capture, enumeration, and characterization of exfoliated tumor cells in urine. Sensitivity (53.3% vs. 40.0%) and specificity (100% vs. 95.8%) of microfilter cytology were higher than standard cytology. Miyake et al. (2021) created a high-throughput detection device for hexylaminolevulinate-mediated fluorescent voided urine cytology, and this novel approach was found to have significantly higher sensitivity compared with standard cytology (63% vs. 29%).

Over the last few decades, numerous studies have tried to explore novel techniques to detect urothelial carcinoma by analyzing protein and nucleic acid in urine. Several methods such as bladder tumor antigen, FISH, and NMP22 have been approved by the FDA. However, clinical application of these markers is still rare due to the lower specificity compared with UCT and the low reproducibility. None of these markers have been accepted for diagnosis or follow-up in daily practice or clinical guidelines (Babjuk et al., 2022). More recently, several detection platforms based on high-throughput sequencing have been developed, such as CxBladder (Kavalieris et al., 2016) and Xpert (Valenberg et al., 2019) (multigene expression markers); Bladder EpiCheck (Mancini et al., 2020), UriFind (Ruan et al., 2021), and utMeMA (Chen et al., 2020) (DNA methylation markers); and UroCAD (Chromosomal instability) (Zeng et al., 2020). These novel markers were reported to have both high sensitivity and specificity and NPV. Valenberg et al. (2019) developed the Xpert Bladder Cancer Monitor (Xpert) and measured five mRNA targets (ABL1, CRH, IGF2, UPK1B, and ANXA10) that are frequently overexpressed in BC. The overall sensitivity was 74% (63% for LG, 83% for HG) and specificity was 80%. Ruan et al. (2021) developed and validated a urine-based PCR DNA methylation assay for early detection of BC, which showed a sensitivity of 88.1%–91.2%, a specificity of 89.7–85.7%, and superior sensitivity in detecting low-grade (66.7%–77.8%) and Ta tumors (83.3%). Compared with these detection platforms based on the high-throughput sequencing, nano-cytology showed similar sensitivity for low-grade (79.1%) and Ta tumors (78.3%). However, these liquid biopsy methods are still not widely used in clinical practice; further studies with a larger sample size in multiple centers and longer follow-up are warranted.

Nanotechnology has widely been developed for the management of cancers from diagnosis to treatment (Tomlinson et al., 2015). However, nanotechnology applied on the noninvasive detection of BC is limited. Eissa et al. (2014) described a gold nanoparticle assay in which target RNA was purified using magnetic nanoparticles (Fe_2_O_3_) and then detected with standard molecular techniques. This approach reached a sensitivity of 88.5% and a specificity of 94%. Hyaluronanase was detected in urine based on the agglomeration characteristics of cationic gold nanoparticles to diagnose BC in 40 participants; this study showed that the sensitivity was 90% and the specificity was 80.8% when the threshold value was 93.5 μU/ng protein (Nossier et al., 2013). Nossier et al. (2016) developed a simple colorimetric gold nanoparticle assay for the detection of urinary total gelatinase activity (MMP-2 and MMP-9) to differentiate BC patients, and resulted in a sensitivity of 87.5% and a specificity of 86.4%.

Based on the fact that the tumor cell membrane is negatively charged, nanoparticles with positive electricity are more conducive to bind to the cell membrane (Chen et al., 2016; Hla et al., 2021). Inspired by unlike electric charges attract, we utilized the positively charged mNPs to capture tumor cells in urine. The morphology of low-grade tumor cells is not much different from normal urothelium. As a result, it is difficult to identify low-grade tumor cells with plenty of background cells, which leads to lower sensitivity compared to high-grade tumor cells in standard UCT (Barkan et al., 2016). The Nano-cytology method could identify tumor cells by the nano-ring phenomenon and enrich tumor cells and reduce the influence of a large number of background cells in exfoliated cell slides, thus improving the reading efficiency and accuracy of cytopathologists.

Limitations of the present study should be noted. First, mNPs are synthesized and provided by the laboratory we worked with, and nano-cytology is currently only available in our hospital. As a result, this is a single-center study. Second, only a small number of urine samples were included in the present study, and the value of nano-cytology should be confirmed *via* a larger cohort and multicenter study in the future. Third, Nano-cytology was the combination of the “nano-ring” phenomenon and the cytology test of the mNP-enriched cells. Compared with UCT, the cytology test of mNP enrichment lost some of the urine exfoliated cells. Fourth, nano-ring is essential to capture tumor cells, but we have found that mNPs could also be absorbed by few leukocytes such as granulocytes in a previous study (Chen et al., 2016). In the present study, five patients with a false-positive result (nano-ring phenomenon on white blood cells) were found to have a high level of white blood cells in urine (at least 280 cells/HPF). Predictably, the diagnostic accuracy can be influenced in patients with severe urinary tract infection, and nano-cytology should be conducted after infection is under control. Fourth, although mNPs are specific for tumor cells, they cannot capture tumor cells less than 50/50 ml; the lack of sufficient exfoliative cells in urine from patients may be the major technique limitation. So, the combination of nano-cytology and UCT will help to improve detection sensitivity. Fifth, we did not compare the diagnostic performance of nano-cytology with FISH assay, because urine sample collected at one urination is not enough for three urine tests, and urine collected at different times may further introduce bias.

## Conclusion

Compared with standard UCT, the Nano-cytology assay showed significantly improved sensitivity and comparable specificity for detecting BC. Nano-cytology may be a promising novel approach adjunct to cystoscopy for diagnosis of BC in clinical practice.

## Data Availability

The original contributions presented in the study are included in the article/supplementary material. Further inquiries can be directed to the corresponding authors.
